# Effect of Case Variation on Mastoidectomy Training Using 3D‐Printed Temporal Bones Models—A Prospective, Randomized Trial

**DOI:** 10.1002/ohn.70358

**Published:** 2026-08-03

**Authors:** Adam Omari, Andreas Frithioff, Anders Nøhr, Mads S. Sørensen, Steven A. W. Andersen

**Affiliations:** ^1^ Department of Otolaryngology–Head & Neck Surgery and Audiology, Rigshospitalet, Copenhagen Hearing and Balance Center Copenhagen University Hospital Copenhagen Denmark; ^2^ Institute for Cellular and Molecular Medicine (ICMM), Faculty of Health and Medical Sciences University of Copenhagen Copenhagen Denmark; ^3^ Copenhagen Academy for Medical Education and Simulation (CAMES) RegionH Copenhagen Denmark; ^4^ Department of Clinical Medicine, Faculty of Health and Medical Sciences University of Copenhagen Copenhagen Denmark

**Keywords:** 3D‐printing, cognitive load, instructional design, learning curve, mastoidectomy, simulation based training, temporal bone, training

## Abstract

**Objective:**

To investigate effects of repeated training with anatomically varied versus identical 3D‐printed models on skill acquisition, cognitive load, and skill retention in novice mastoidectomy trainees.

**Study Design:**

A prospective, randomized, controlled, educational trial conducted April to September 2024 including a 3‐month retention test.

**Setting:**

Simulation center at a single academic teaching hospital.

**Methods:**

Twenty‐four novice medical students were randomized 1:1 into 2 groups: The Identical Models (IM) group practiced repeated procedures on identical 3D‐printed temporal bone models (control) and the Variable Model (VM) group practiced on a varied selection of models (intervention). Trainees completed 10 anatomical mastoidectomy procedures with posterior tympanotomy followed by 2 retention test procedures after 3‐months of nonpractice. The primary outcome was performance assessed with 25‐point modified Welling Scale. The secondary outcome was cognitive load estimated by relative reaction time measured on a secondary task.

**Results:**

At end‐of‐training, the VM group scored 20.6 points (95% CI [19.2‐22.0]), outperforming the IM group who scored an average of 18.2 points (95% CI [16.7‐19.6], *P* = .02). At 3‑month retention testing, the VM group's scores decayed by a mean of 5.6 points on the repeated model (95% CI [−7.2 to−4.0], *P* < .001) and of 3.7 points when introduced to a new model (95% CI [−5.2 to−2.1], *P* < .001). The IM group showed no decay thereby demonstrating better skill retention which aligned with reduced relative reaction time (cognitive load) during training.

**Conclusions:**

Educators can optimize mastoidectomy skills acquisition and retention by instructional design that prioritize repeated practice on anatomically identical models until basic skills have been acquired.

Proficiency in mastoidectomy depends on excellent technical surgical skills and comprehensive knowledge of the temporal bone (TB) anatomy. Novice trainees require extensive training to become proficient^1^ but providing such training is increasingly difficult using traditional cadaver dissection alone given the scarcity, legal hurdles, and high costs.^2^, ^3^ Consequently, simulation‐based training using three‐dimensional (3D)‐printed models is rising in popularity^4^ and continues to improve in realism and cost‐effectiveness as the printing technology matures.^5^

The simple drilling exercise of the traditional TB course forms a universal learning routine based on consensus and limited by biological and logistic constraints. The newer training technologies provide educators with better possibilities for optimizing *instructional designs*—the specific tools and strategies used to best plan, validate, and implement evidence‐based training.^6^ For example, they can provide easy access to unlimited repetitions on varied (as is the case with cadaveric TBs) or identical anatomies in VR or 3D print.

However, surgical educators trying to develop new simulation‐based training methods face limited evidence on instructional design.^6^ Studies on mastoidectomy training using 3D‐printed models mostly rely on outcomes based on subjective expert and/or trainee opinion (face validity).^4^ The few studies that include objective performance assessment are based on a maximum of only three identical training procedures.^7^, ^8^, ^9^ None have explored additional repetitions or the role of anatomical variation of the 3D‐printed models, despite both being instructional design elements generally considered best‐practices.^6^

Altogether, little is known on the learning curve of repeated practice of mastoidectomy using 3D‐printed models despite this is crucial for the training curriculum. Establishing the learning curve allows for evidence‐based decisions on instructional design to determine *if* additional repetition is needed and *when* to add new challenges such as anatomical variation or other modalities such as cadaver practice or supervised real surgery to better sustain learning.^6^, ^10^ Optimized simulation allows trainees to learn as much as possible before supervised real surgery, moving the initial learning phase away from patients and into a safe simulated environment.

The development of psychomotor skills presents a major challenge in the initial acquisition of surgical skills.^11^ Cognitive load (CL) theory argues that the capacity of working memory and information processing is limited and can be overloaded when the cognitive demands exceed the trainee's cognitive capacity.^12^ When novice trainees need to integrate the complex information and motor skills coordination, the introduction of anatomical variation could thus be counterproductive to early acquisition of skills and skills retention. Instructional designs that promote cognitive engagement^6^ and reduce unnecessary CL^13^ seem to be beneficial for learning processes, as it is critical to prevent trainee skill acquisition from stalling due to either lack of challenge or cognitive overload.

The objective of this study was to investigate the effects of repeated training with anatomically varied versus identical 3D‐printed models on skill acquisition, CL, and skill retention in novice mastoidectomy trainees. We hypothesized that introducing anatomical variation would initially slow skill acquisition on the learning curve yet enhance the transfer of skills to new anatomical variations. This is important as educational resources and clinical training time are limited.^14^

## Methods

### Study Design and Participants

This was a prospective, randomized, controlled study of an educational intervention at the simulation center of an academic teaching hospital (April‐September 2024). Medical students, naïve to the mastoidectomy procedure, from the University of Copenhagen were recruited via student fora; the sole exclusion criterion was prior TB simulation experience (Table 1).

**Table 1:** Participant Characteristics

	Varied Models (VM) Group	Identical Models (IM) Group
No. of trainees (%)	12/24 (50)	12/24 (50)
Age (years), mean (SD)	24.0 (2.0)	26.9 (6.3)
Sex, no. (%)	Male 4 (33) – female 8 (67)	Male 6 (50) – Female 6 (50)
Years of study, mean (SD)	3.8 (1.3)	3.8 (2.0)
Handedness, no. right (%)	10/12 (83)	12/12 (100)
Interest in surgery 7‐point Likert scale, Median (IQ‐range)	6.5 (2.0)	6.0 (2.0)

Abbreviations: IQ‐range, inter‐quartile range; SD, standard deviation.

### Randomization

The study compared 2 groups randomized to training on either (1) a series of identical 3D‐printed models (IM group, control) or (2) a series of variable 3D‐printed models (VM group, intervention). Participants were randomized 1:1 using an online tool.^15^ Drop‐outs were replaced by new trainees assigned to the same group who completed the full training protocol from the start. Flow chart is provided in Figure 1.

**Figure 1:**
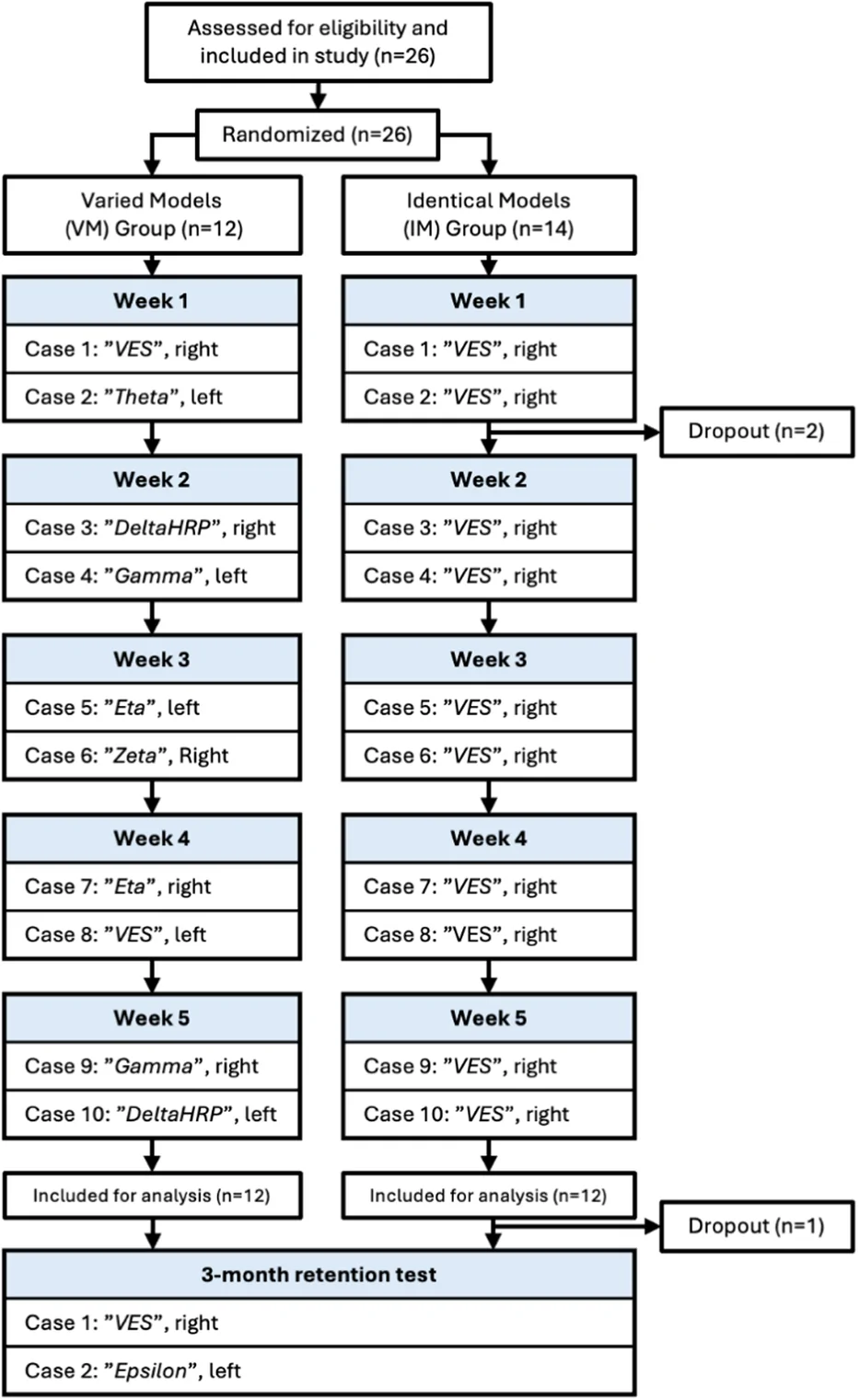
Study flow diagram. The study flow diagram outlines the distributed training and retention test.

### 3D‐Printed TB Models

The 3D‐printed models were manufactured in‐house based on our existing high‐detail library of cone‐beam CT (CBCT) datasets^16^ or cryosections.^17^ The models were printed in a “natural”‐colored, slightly translucent, ABS filament on X1‐Carbon (Bambu Lab) using the default nozzle (0.4 mm), 0.2 mm slice thickness, infill of 85%, and printed in a cranio‐to‐caudal orientation (Supplemental Figure S1, available online). All other settings were left on ABS printer defaults. Print time was 2 to 2.5 hours per specimen and approximate filament costs were ~1.5 USD/model. Post‐processing consisted of removing scaffolds, coloring the sigmoid sinus and dura using acrylic paint, and inserting a colored wire into the fallopian canal to represent the facial nerve (Figure 2).

**Figure 2:**
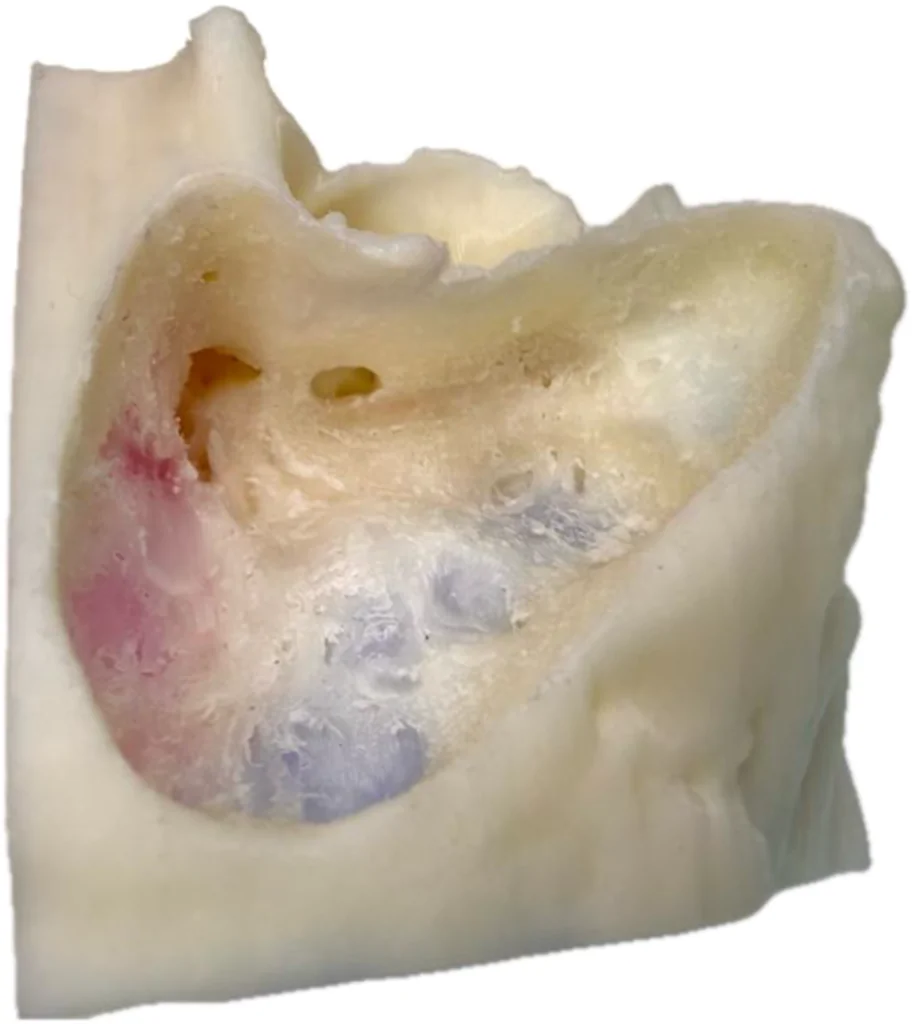
Drilled 3D‐printed model. Drilled 3D‐printed model displaying a trainee's mastoidectomy and posterior tympanotomy. Visible landmarks include the incus short process, facial nerve contour, dura, sigmoid sinus, and round window niche.

### Study Procedures and Intervention

In each of the 5 training sessions, participants performed 2 mastoidectomies with posterior tympanotomy with a 90‐minute time limit per procedure. This duration ensured sufficient drilling time based on time limits previously applied to novices.^18^ Sessions were spaced a week apart to ensure distributed training. The IM group performed training on identical, right‐sided 3D‐printed models based on the VES‐dataset^17^ and the VM group trained on 5 different anatomies and a mix of right and left models from the OpenEar datasets,^16^ ensuring no identical cases during training. To estimate CL, participants performed a secondary reaction time task during the drilling procedure. Measurements were taken in sets of three at baseline (before each drilling procedure) and at three intervals (*t* = 10, 30, and 50 minutes) during the drilling procedure. RTs were recorded with a commercial reaction timer (American Educational Products, LLC), which captured the interval (in 1/100 s increments) between an auditory stimulus (beep) and participant response (pressing a foot switch). CL was estimated by calculating the RT during each procedure as a multiple of the pretraining baseline,^1^ that is, the proportional increase in RT (relative reaction time, RRT), which reflects spare cognitive capacity (faster response = lower RRT ≈ lower CL).

Prior to the first session, participants provided written consent, supplied demographic information, and received basic instruction on anatomy, drill handling, and microscope use. A step‐by‐step manual and an exemplary drilled model were available throughout the study except for the retention test procedures. Faculty intervened only for equipment issues or general questions. Equipment included otosurgical drills (Bien Air, Biel/Bienne), a typical array of drill bits, and operating microscope (Carl‐Zeiss OPMI Pico) (Supplemental Figure S2A and SB, available online). Plastic drill dust was removed manually using compressed air without suction or irrigation.

Three months after completing the training procedures, participants completed the retention test, which consisted of two procedures: one right‐sided (identical to the IM model) and one novel left‐sided model (new to all participants).

### Outcomes

The primary outcome was final‐product performance using the modified Welling Scale (WS), which is a 26‐item, binary assessment instrument.^19^ The maximal WS score was reduced to 25 items (for a maximum of 25 points) because the 3D‐printed models did not include chorda tympani. All final products were assessed independently by 2 raters (MSS and SA) with extensive experience in mastoidectomy performance assessment. The raters were blinded to participant ID, and procedure number as models were coded and mixed for random assessment order. This effectively blinded groups to the extent possible, though morphologic differences in the TB shapes themselves theoretically could inform on group assignment. The secondary outcome was CL, estimated using secondary‐task reaction time (RT)^20^ in line with previous studies.^21^, ^22^, ^23^

### Sample Size and Statistics

Sample size calculations are not well defined for study designs using repeated measures,^24^ owing to correlation among pairs of repeated measurements as intra‐individual correlation from two or more successive measurements should be accounted for.^25^, ^26^ We determined a total of n = 24 trainees should be included based on comparable studies^27^ and satisfies the minimum requirements for the robustness of the linear mixed‐effects models used.^28^

Data analysis for all participants relied mainly on linear mixed models (LMM) to account for the repeated measures design, following the recommendations by Leppink.^26^ For training scores, the final LMM included group (IM or VM), procedure number (1‐10) and rater (SA and MS) as fixed factors. For CL, the final LMM included group, procedure number, measurement sequence (1‐3) as fixed factors. The group‐procedure number interaction was included in both models, as it was significant for reaction time and approached significance for training scores. All mean scores and 95% confidence intervals reported were estimated marginal means from LMM analyses. Between‐group comparisons were conducted using independent samples *t*‐tests where applicable. To visualize learning curves, a LOESS smoothing function^29^ with span parameter 0.8 was applied to smooth curves to the nearest data points to balance trend preservation and noise reduction. The resulting scores at each procedure are connected to form a curve representing the estimated mean performance, while the surrounding shaded regions represent the 95% confidence intervals (CI). The slope of the line indicates the rate of learning across all 10 consecutive training procedures. SPSS Statistics Software version 28.0 (IBM) was used along with R Studio version 1.3.1093 (RStudio, PBC). *P* < .05 were considered statistically significant.

This study was conducted and reported in accordance with the Consolidated Standards of Reporting Trials (CONSORT) guidelines (Supplemental Figures S3 and S4, available online).^30^

### Ethics

This study was conducted in accordance with the Helsinki Declaration. Participation was voluntary and participants were offered no study credits or compensation for participation. Written and oral information and informed consent were ensured before enrolment.

## Results

Twenty‐six participants were enrolled in the study. Two participants dropped out after their first session and were replaced; another participant dropped out after initial training (10 procedures) and did not complete retention test procedures. These participants all withdrew because of time constraints. Twenty‐four completed training and were included for analysis with 12 participants per group (Table 1). A total of 286 procedures (3D‐printed models) were available for final‐product analysis.

### Training Performance

In the very first procedure, the IM group performed slightly better (10.1 points; 95% CI [8.7‐11.5]) than the VM group (8.0 points; 95% CI [6.6‐9.5]) (LMM, *P* = .046) (Figure 3). Both groups demonstrated improvement in final‐product performance over the course of the 10 training procedures as visualized in the learning curves (Figure 3). The IM group showed rapid early performance increase but reached a plateau around procedure five, achieving a mean performance score of 18.2 points (95% CI [16.7‐19.6]) at the end of training (ie, procedure 10). In contrast, the VM group improved slowly and did not exhibit a clear performance plateau although the learning curve slope became less steep after procedure five. At the end of training, the mean performance of the VM group was 20.6 points (95% CI [19.2‐22.0]), which was higher than the IM group (*P* = .02). Item analysis revealed that the VM group outperformed the IM group especially on performance related to the sigmoid sinus and tegmen tympani where the IM group's skills seemed to plateau during training (Figure 4).

**Figure 3:**
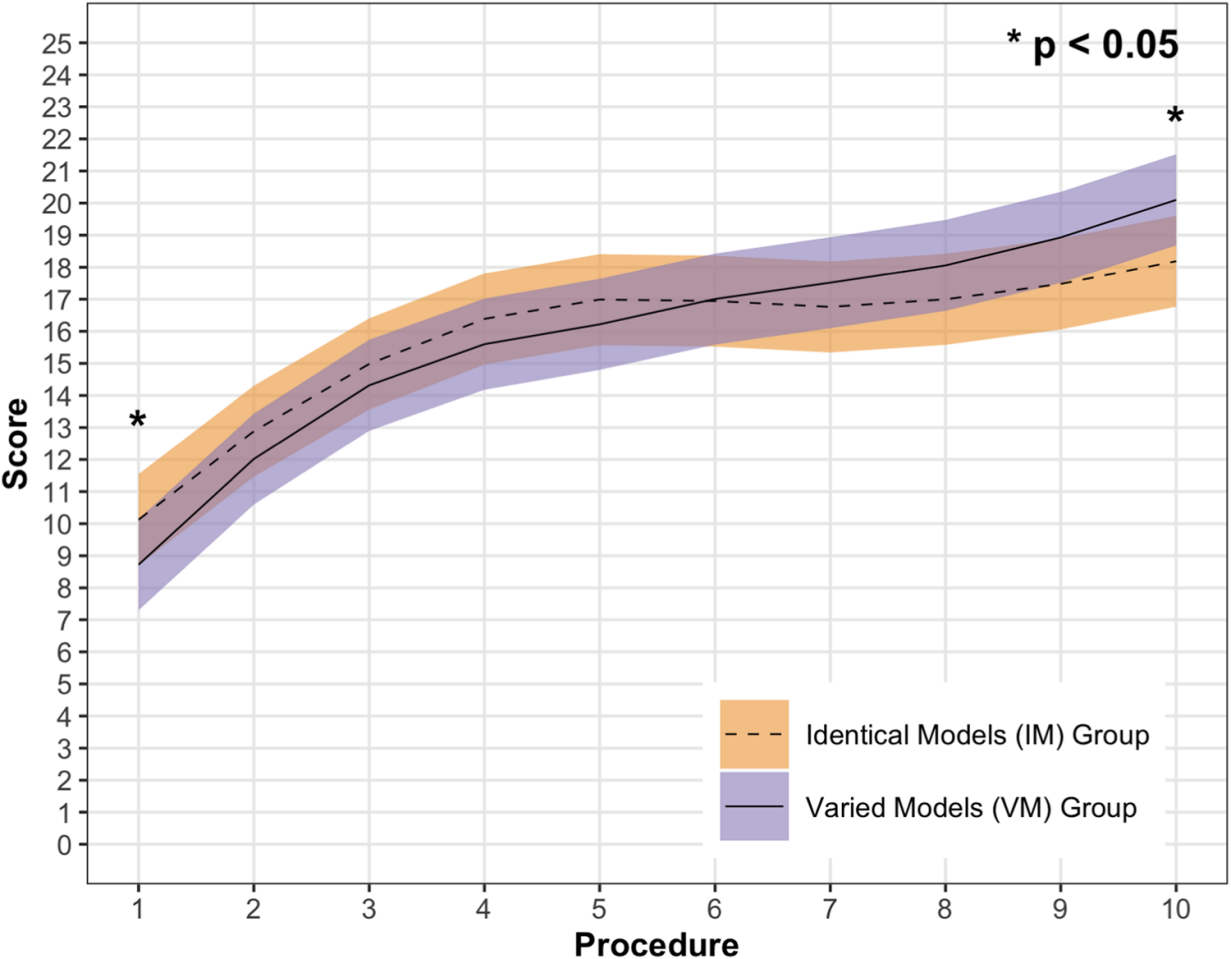
Learning curve. Rate of learning over 10 consecutive training procedures. Solid lines show estimated mean Welling Scale scores (0‐25) per group. Results display LMM‐derived means as absolute values; with shaded 95% confidence intervals. [Correction added on 3 September 2026, after first online publication: Words “maximum percentages” have been changed to “absolute values.”]

**Figure 4:**
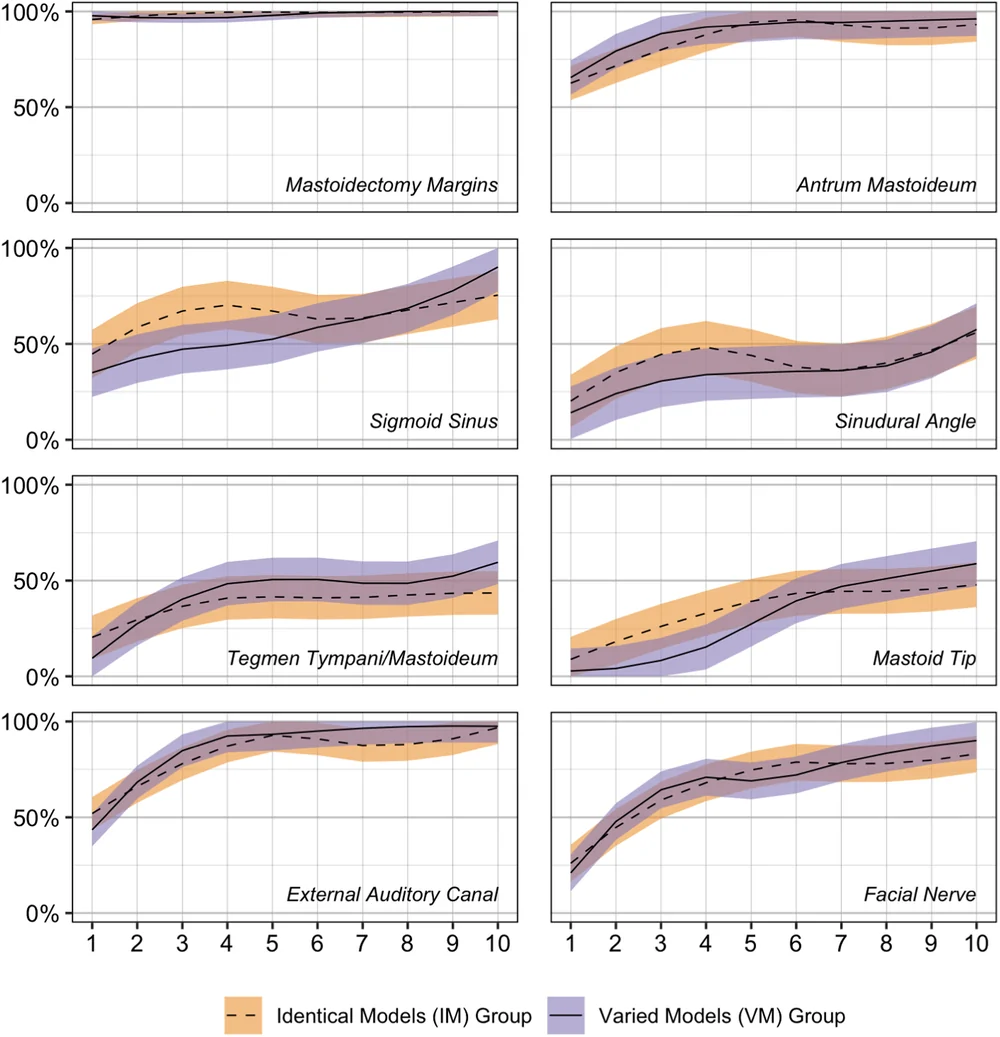
Learning curve for specific domains. Line slopes illustrate learning across eight domains (grouped from 25 individual Welling scale items). Results display LMM‐derived group mean scores as a percentage of the maximum; shaded 95% confidence intervals.

### CL

The RRT, reflecting CL, was slightly higher at baseline in the IM group (1.81; 95% CI [1.72‐1.91]) compared to the VM group (1.66, 95% CI [1.56‐1.76]) (LMM, *P* = .03) (Figure 5). The IM group showed a steady decline in RRT and ended at 1.43 (95% CI [1.33‐1.54]) compared to their starting point of 1.81 (mean difference = 0.38, *P* < .001). In contrast, the RRT remained largely unchanged in the VM group and ended at 1.63 (95% CI [1.52‐1.73]) (mean difference = 0.03, *P* = .64) (Figure 5).

**Figure 5:**
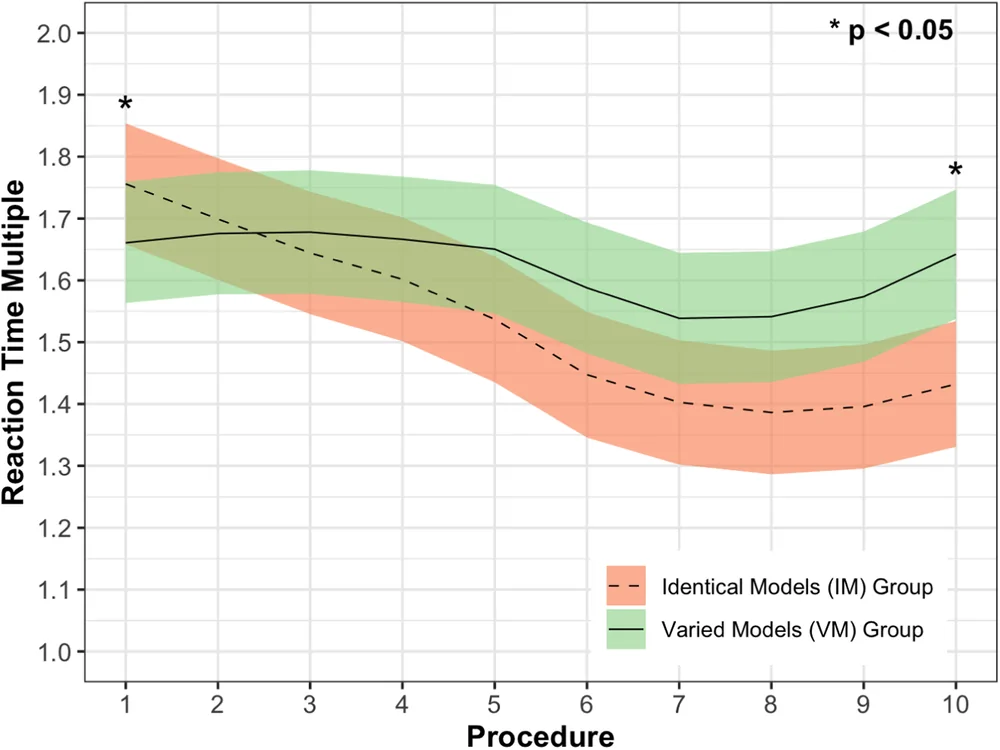
Reaction time during training*.* Means with 95% confidence intervals. Reaction times displayed as a multiple of the baseline measurements taken immediately before start of each procedure.

### Retention Performance

For the retention test procedures, only the VM group demonstrated skills decay compared to their end‐of‐training performance. For the first retention procedure, the VM group scored 5.6 points lower (95% CI [−7.23 to −3.99], *P* < .001), whereas the IM group showed no difference with a mean increase of 0.8 points (95% CI [−0.83 to 2.49], *P* = .29) (Figure 6A). Similar results were found for the second procedure, which was completely new to both groups: the VM group scored 3.7 points lower than their end of training procedure (95% CI [−5.22 to −2.09], *P* < .001) whereas the IM group showed no apparent skills decay with a mean increase of 0.1 points (95% CI [−1.52 to 1.79], *P* = .87) (Figure 6B).

**Figure 6:**
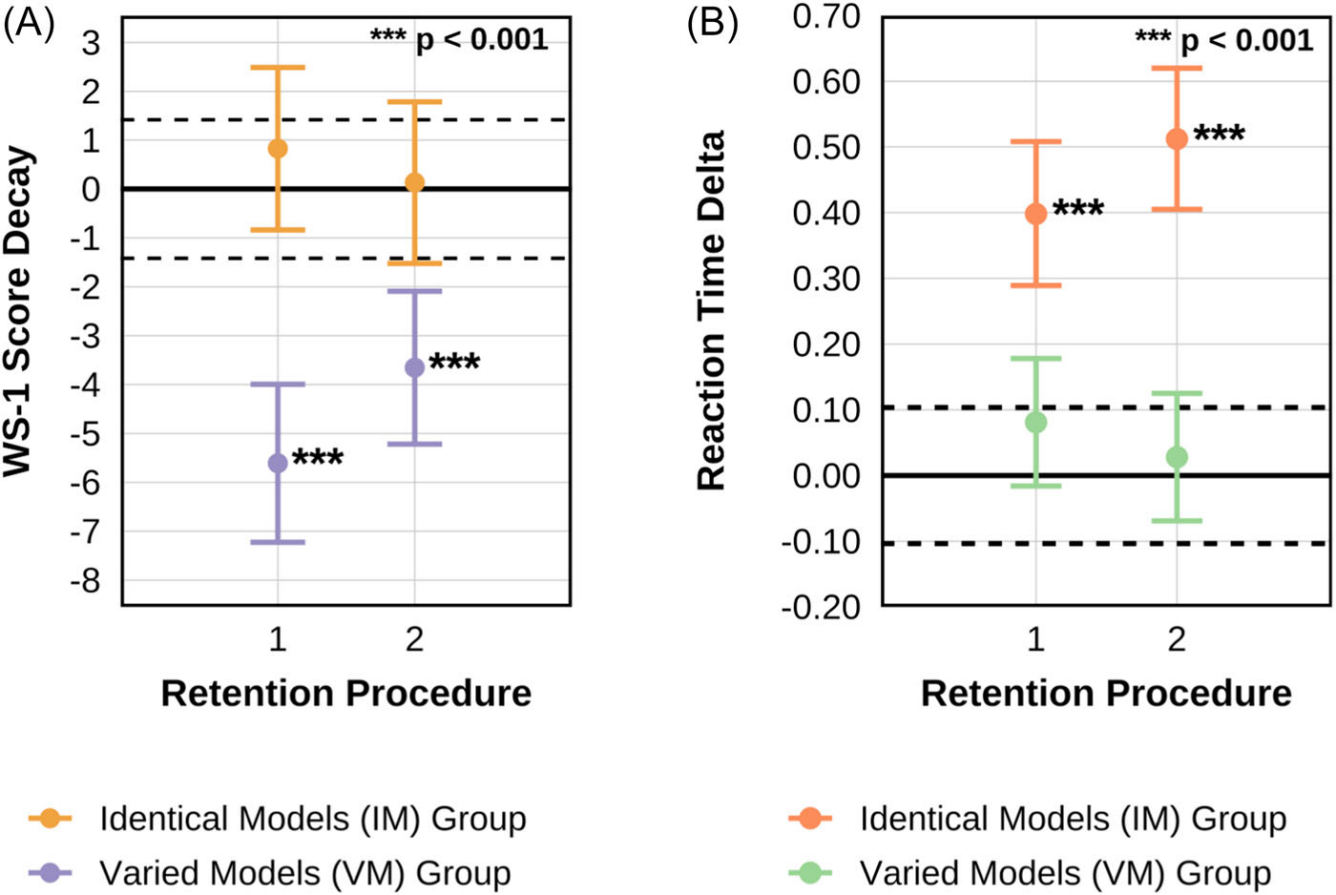
(A) Three‐month skill retention. Solid lines show end‐of‐training group means (procedure 10 = baseline); dotted lines show 95% confidence intervals. *X*‐axis: retention procedure count. *Y*‐axis: mean WS score decay from end‐of‐training baseline adjusted for procedure duration. (B) Three‐month reaction‐time change. Solid lines show end‐of‐training (procedure 10 = baseline) mean relative reaction time (RRT); dotted lines show 95% confidence intervals. *X*‐axis: retention procedure count. *Y*‐axis: mean RRT change (seconds) in comparison to end‐of‐training baseline.

In contrast to the final product performance, the RRT was stable from end‐of‐training to retention for the VM group (first retention procedure 1.71, 95% CI [1.61‐1.80], *P* = .27; second retention procedure 1.65, 95% CI [1.56‐1.75], *P* = .70). The IM group had a higher RRT, and thereby a higher CL, compared to their end of training measurements (first retention procedure 1.83, 95% CI [1.72‐1.94], *P* < .001; second retention procedure 1.95, 95% CI [1.84‐2.05], *P* < .001).

## Discussion

Simulation‐based training using 3D‐printed TB models followed traditional learning curves across ten procedures with anatomical variation modifying learning curves, retention, and CL. Training on identical models led to an early learning curve plateau, but skills were retained long‐term and transferred directly to a high performance on a completely new model. In contrast, training on models with anatomical variation led to continued skills improvement without an apparent plateau and with a higher end‐of‐training performance but at the cost of a substantial skill decay after three months of nonpractice.

The results highlight that an early plateau is not in fact evidence of ineffective training, as sustained practice to reach a plateau on the learning curve seems important for consolidating acquired skills in long‐term memory. Optimizing training for long‐term skill retention ensures that trainees have core competencies ready when they get the opportunity to practice on cadavers or perform supervised surgery. This also highlights the need for educational studies to evaluate long‐term skill retention to fully understand the impact of instructional design.^31^

An effective instructional design should facilitate the transfer of acquired domain‐specific skills from working memory to long‐term memory where it can be stored and used to govern future actions.^32^ Medical education theory suggests that instructional designs with anatomical variation is superior^33^ and among the best‐practice features in effective simulation training designs.^6^ Systematic reviews on instructional designs emphasize the pressing need to clarify what works, for whom, and under which circumstances.^6^, ^31^ Our results demonstrate that anatomical variation of 3D‐printed simulation models should be used sparingly for initial training of novice trainees. When introduced too early, it seems to impair long‐term learning outcomes, suggesting that its effectiveness is heavily moderated by task complexity and learner experience. CL theory^12^ can provide a useful framework for understanding how the instructional design affected learning in this study. Learning depends on the balance of three types of load: *Intrinsic load* is the unavoidable complexity of the mastoidectomy procedure itself, *extraneous load* is the extra effort created by inefficient instructional design, and *germane load* is the remaining cognitive bandwidth left for mental schema formation to “learn” the procedure.^12^ Therefore, training on identical models reduced intrinsic load by removing the need to constantly calibrate to new anatomical variation on every procedure. This frees up bandwidth to better process germane load to consolidate freshly acquired skills into long‐term memory. Educators must consider the timing of when to introduce anatomical variation in training to balance continuous skills acquisition and lasting retention. A staged training curriculum could be a solution where educators implement a series of identical 3D‐printed models to cement basic skills automation and decrease intrinsic CL before introducing case variation. Still, it remains unknown how well skills transfer to the clinical setting. Based on our results, we hypothesize that early clinical training should aim to simplify objectives and moderate exposure to anatomical variation. One option is trainees start with repetition of simple, lower‐risk steps of the mastoidectomy such as defining the mastoid margins and saucerising the cortical bone. As trainees gain proficiency, educators can gradually introduce subsequent higher‐risk steps where safe navigation of anatomical variation becomes increasingly paramount.

A few previous studies have explored training on 3D‑printed TB model and focused on skills transfer to cadaver dissection. In one study, a single training performance raised cadaveric dissection scores by more than four WS points compared with no training.^9^ We have previously reported in another study that three consecutive training performances on 3D‐printed models yielded a 29% improvement of cadaver dissection compared with time‐equivalent VR simulation training.^8^ We also recently demonstrated the importance of aligning simulation fidelity with the trainee proficiency, by showing training on two generic educational 3D‐printed models yielded a 9% improvement of cadaver dissection compared with cadaver‐specific 3D‐printed model training.^34^ So, while educators can rely on measurable benefits in cadaver transfer which perhaps explain their positive sentiment,^2^ questions still remained on how much trainees could expect to develop on the simulation platform before they should advance to cadavers or real supervised surgery. Therefore, this study importantly sets educators expectations on trainee development, offers an evidence‐base for when to progress and why anatomical variation should be moderated, and does so while examining skills retention to reflect timing issues of training in the real world. The substantial inter‐individual variability in performance we found further highlights the need for individualized instructional designs, such as mastery learning approaches,^35^, ^36^ in which trainees progress is based on demonstrated proficiency at a predefined performance level.^36^, ^37^ The most comparable study designs use VR simulation training. One study by our group found that case variation did neither benefit or impair novices during training, retention testing, nor in skills transfer to a 3D‐printed model.^5^ Another study found that drilling of 12 varied cochlear implant anatomies did not result in better performance except for a small benefit for the facial nerve and round window.^38^ The learning curve in VR has been shown to plateau at an insufficient performance level^39^─even with case variation,^27^, ^39^ guidance and feedback mechanisms.^40^, ^41^ Because of different task conditions between studies, our results alone cannot substantiate a claim that 3D‐printed models are superior to VR simulation. Future randomized trials are needed to compare VR head‐to‐head or in synergy with 3D‐printed models to define the optimal mix and sequencing of the two modalities.

A strength of this study was the randomized, controlled design, and the blinded performance assessment using an assessment tool with extensive validity evidence,^42^ supporting a high internal validity. Further, the repeated‐measures design allows robust statistical power despite the relatively small number of participants in each group. Finally, the study is extensive in that each participant drilled and was evaluated on the largest number of 3D‐printed models reported to date. Limitations include that participants were novice medical students, which may limit generalizability to more experienced trainees. Next, while our 3‑month retention test extends beyond intervals used in comparable studies,^27^ it may still remain shorter than the typical wait for real clinical practice opportunities. Finally, while the FDM‐based 3D‐printed models offered satisfactory anatomical detail and low production cost, they lack the finest details, in particular moveable ossicles and the chorda tympani which could limit transferability to cadaveric and real‐life performance. We mitigated some of these limitations by use of neutral‐colored ABS filament to maximize heat resistance, translucency, and preservation of drill feel. This choice also ensured a simple, cost‐effective (~1.5 USD/model) workflow designed to maximize accessibility for widespread training.

## Conclusion

This study demonstrates that for novices, repeated practice on identical 3D‐printed TB models improves skill acquisition, reduces CL, and preserves long‐term skills retention. In contrast, anatomical variation promotes continuous learning but impaired skills retention and transfer to a novel anatomical variant, likely due to sustained induction of a higher CL during training. Surgical educators should implement a staged training curriculum for novices, using identical models to establish basic proficiency and only then introduce variation to sustain the positive slope on the learning curve. This provides educators with a clear, evidence‐based instructional design for mastoidectomy training curricula to better prepare trainees for cadaver dissection and supervised real surgery.

## Author Contributions

**Adam Omari**, protocol, synthesis, drafting; **Andreas Frithioff**, Protocol, drafting. **Anders Nøhr**, Protocol, drafting. **Mads S. Sørensen**, synthesis, drafting; **Steven A. W. Andersen**, protocol, synthesis, drafting.

## Disclosures

### Competing interests

Dr Andersen has consulted for Ambu A/S (not related to this study).

### Funding source

Novo Nordisk Foundation (grant number #0084895).

## Supporting information


**Supplementary figure 1.** A batch of newly 3D‐printed identical temporal bones on a print bed; **Supplementary figure 2A.** Drill and microscope setup used by the novice trainees; **Supplementary figure 2B.** Fixation of the 3D‐printed temporal bone model. Visual cues are seen such as the sigmoid sinus (blue color) and the facial nerve inside the fallopian canal (slightly dark red color); **Supplementary figure 3:** CONSORT 2025 Flow Diagram. Flow diagram of the progress through the phases of a randomised trial of two groups (that is, enrolment, intervention allocation, retention test, and data analysis; **Supplementary Figure 4:** CONSORT 2025 ITEM Checklist.
